# Improving Selection for Sentinel Lymph Node Biopsy Among Patients With Melanoma

**DOI:** 10.1001/jamanetworkopen.2023.6356

**Published:** 2023-04-19

**Authors:** James R. Miller, Serigne N. Lo, Mehdi Nosrati, Jonathan R. Stretch, Andrew J. Spillane, Robyn P. M. Saw, Kerwin F. Shannon, Omgo E. Nieweg, Sydney Ch’ng, Kevin B. Kim, Stanley P. Leong, John F. Thompson, Richard A. Scolyer, Mohammed Kashani-Sabet

**Affiliations:** 1 Center for Melanoma Research and Treatment, California Pacific Medical Center and Research Institute, San Francisco; 2 Melanoma Institute Australia, The University of Sydney, Sydney, New South Wales, Australia; 3 Faculty of Medicine and Health, The University of Sydney, Sydney, New South Wales, Australia; 4 Department of Melanoma and Surgical Oncology, Royal Prince Alfred Hospital, Sydney, New South Wales, Australia; 5 Department of Breast and Melanoma Surgery, Royal North Shore Hospital, Sydney, Australia; 6 Charles Perkins Centre, University of Sydney, Sydney, New South Wales, Australia; 7 NSW Health Pathology, Sydney, New South Wales, Australia; 8 Tissue Pathology and Diagnostic Oncology, Royal Prince Alfred Hospital, Sydney, New South Wales, Australia

## Abstract

**Importance:**

Refining eligibility guidelines may identify more appropriate patients to undergo useful medical procedures.

**Objective:**

To improve cost-effectiveness in selecting patients with melanoma for sentinel lymph node biopsy (SLNB).

**Design, Setting, and Participants:**

This hybrid prognostic study/decision analytical model was conducted among patients with melanoma who were eligible for SLNB at 2 melanoma centers from Australia and the US from 2000 to 2014. Participants consisted of 2 cohorts of patients with melanoma undergoing SLNB and a cohort of eligible patients without SLNB. Individualized probabilities of SLNB positivity generated by a patient-centered methodology (PCM) were compared with those generated by conventional multiple logistic regression analysis investigating 12 prognostic factors. Prognostic accuracy was assessed by the area under the receiver operating characteristic curve (AUROC) for each methodology and by matched-pair analyses.

**Interventions:**

Triaging appropriate patients to undergo SLNB.

**Main Outcomes and Measures:**

Total number of SLNBs performed (giving total cost) vs number of SLNB-positive outcomes (a measure of effectiveness) was evaluated. Improved cost-effectiveness through judicious patient selection was interpreted as increased numbers of SLNB-positive outcomes achieved, decreased numbers of SLNBs performed, or both outcomes simultaneously.

**Results:**

Among 7331 patients with melanoma, SLNB outcomes were assessed in 3640 Australian patients (2212 males [60.8%]; 2447 aged >50 years [67.2%]) and 1342 US patients (774 males [57.7%]; 885 aged >50 years [66.0%]); 2349 patients eligible for SLNB who did not undergo the procedure were included in the simulation. PCM-generated probabilities achieved an AUROC of 0.803 in predicting SLNB positivity in the Australian cohort and 0.826 in the US cohort, higher than corresponding AUROCs generated by conventional logistic regression analysis. In simulation, adopting many SLNB-positive probabilities as minimally acceptable patient-selection criteria resulted in fewer procedures performed or increased the expected numbers of positive SLNBs. A minimally acceptable PCM-generated probability of 8.7% elicited the same number of SLNBs as historically performed (3640 SLNBs), with 1066 positive SLNBs (29.3%), constituting an improvement of 287 additional positive SLNBs compared with 779 actual positive SLNBs (36.8% improvement). In contrast, adopting a 23.7% PCM-generated minimum cutoff probability resulted in performing 1825 SLNBs, or 1815 fewer SLNBs than the actual experience (49.9%). It resulted in the same expected number of positive results (779 SLNBs), for a 42.7% positivity rate.

**Conclusions and Relevance:**

This prognostic study/decision analytical model found that the PCM approach outperformed conventional multiple logistic regression analysis in predicting which patients would have positive results on SLNB. These findings suggest that systematically producing and exploiting more accurate SLNB-positivity probabilities could improve the selection of patients with melanoma for SLNB compared with using established guidelines, thus improving the cost-effectiveness of the selection process. Eligibility guidelines to undergo SLNB should include a context-tailored minimum cutoff probability.

## Key Points

**Question:**

Can systematically refining eligibility guidelines identify more appropriate patients to undergo useful medical procedures using sentinel lymph node biopsy (SLNB) for melanoma as an illustrative example?

**Findings:**

In this prognostic study/decision analytical model that included 7331 patients with melanoma, a methodology that significantly improved the prognostic accuracy of probabilities of SLNB outcomes was used in 2 distinct populations. Adopting a range of minimally acceptable probabilities made patient selection for SLNB more cost-effective, often with fewer procedures performed and more patients with node-positive melanoma identified.

**Meaning:**

This study found that improved accuracy in predicting SLNB-positivity could enhance cost-effectiveness in selecting patients for SLNB, suggesting that melanoma guidelines for undergoing SLNB should be refined accordingly.

## Teaser

This prognostic study/decision analytical model investigated the use of methodologies that improve expected prognostic accuracy and thus cost-effectiveness in selecting patients with melanoma for sentinel lymph node biopsy.

## Introduction

The substantial cost of health care delivery constitutes a substantial burden on the economies of industrialized nations. Ample evidence points to the magnitude of waste when patients unnecessarily undergo costly medical procedures.^1^ While medical societies develop guidelines to standardize approaches to medical care, these guidelines are mostly consensus based rather than evidence based and are inconsistently applied. They often fail to consider individual patient characteristics and differences among participating institutions. Strict reliance on established guidelines may therefore overlook patients who may benefit from a procedure while selecting some who are unlikely to benefit. Systematically refining guidelines to reflect relevant contextual factors concerning individual patients and institutions could substantially improve the chances of selecting the most appropriate patients.

Melanoma is the third most common malignant neoplasm in Australia^2^ and the fifth most common in the US.^3^ Major advances in melanoma management have resulted from early diagnosis,^4^ identification of regional lymph node micrometastases,^5^ and development of effective systemic therapies.^6,7,8,9^ Identification of lymph node metastases was revolutionized by the sentinel lymph node biopsy (SLNB) technique. SLNB is an important and reliable staging tool, with SLN status emerging as the most powerful prognostic factor for clinically localized primary melanoma^5,10^ and an important criterion for selecting patients with high-risk, SLNB-positive (stage III) and -negative (stage II) melanoma for adjuvant systemic therapy.^11,12,13,14^ While the procedure is generally well tolerated, it can be associated with complications, such as seroma, infection, and lymphedema.

SLNB is typically recommended to patients based on eligibility criteria, including a primary tumor greater than 1.0-mm thick or thinner melanomas with high-risk features, such as ulceration or increased mitotic rate. The procedure identifies approximately 15% to 20% of patients as having node-positive melanoma.^5^ These patients derive most of the procedure’s benefit (beyond the knowledge of node-negative status) given that patients with SLNB-negative melanoma are often treated in the same manner as if they had not undergone SLNB. This suggests that more accurate prediction of SLNB positivity could reduce the number of procedures performed and increase the number of patients with SLNB-positive melanoma identified, thus improving the cost-effectiveness of the selection procedure. Various nomograms have been developed to reflect risk of SLNB positivity.^15,16^ However, whether more accurate probabilistic algorithms could improve cost-effectiveness in selecting patients to undergo SLNB and the extent of this improvement have not been investigated previously, to our knowledge.

We developed several novel statistical procedures, termed patient-centered methodology (PCM), that generate individually tailored probabilities assignable to specific clinical outcomes (eg, by stratifying patient cohorts into subgroups of differing risk for the outcome of interest and subsequently combining the effect of known prognostic factors within each separate subgroup).^17^ PCM has been shown to enable more accurate prediction of (1) survival outcomes in melanoma and breast cancer^17^ and (2) the performance of mitotic rate in predicting prognoses in melanoma.^18^ This study investigated PCM predictions of individualized SLNB positivity compared with other methods and the resulting cost-effectiveness in selection of patients to undergo SLNB.

## Methods

This hybrid prognostic study/decision analytical model follows the Transparent Reporting of a Multivariable Prediction Model for Individual Prognosis or Diagnosis (TRIPOD) and Consolidated Health Economic Evaluation Reporting Standards (CHEERS) reporting guidelines for observational studies (Table 1; eTable 1 in Supplement 1). Analyses were approved by institutional review boards at each institution (Melanoma Institute Australia [MIA] and California Pacific Medical Center) after informed consent was obtained from each participant.

**Table 1.** Characteristics of Australian and US Cohorts

Characteristic	Patients, No. (%)		*P* value
	Australian (N = 3640)	US (N = 1342)	
Sex			
Females	1428 (39.2)	568 (42.3)	.05
Males	2212 (60.8)	774 (57.7)	
Age >50 y	2447 (67.2)	885 (66.0)	.41
Ulceration	995 (27.5)	262 (22.2)	<.001
Positive SLNB	779 (21.4)	237 (17.7)	.004
TIL grade			
Grade category			<.001
0	1619 (44.5)	NA	
1-3	2021 (55.5)	NA	
Absent	NA	225 (16.8)	
Nonbrisk	NA	373 (27.8)	
Brisk	NA	167 (12.4)	
Not available	NA	577 (43.0)	
Regression			
Late	275 (7.6)	NA	Not comparable
Absent	3034 (83.3)	NA	
Intermediate	331 (9.1)	NA	
No	NA	818 (61.0)	
Partial	NA	18 (1.3)	
Extensive	NA	104 (7.7)	
Not available	NA	402 (30.0)	
Lymphatic invasion			
Absent	3311 (90.9)	852 (63.5)	.002
Present	221 (6.1)	87 (6.5)	
Not available	108 (3.0)	403 (30.0)	
Microsatellites			
No	3219 (88.4)	728 (54.2)	.66
Yes	176 (4.9)	36 (2.7)	
Not available	245 (6.7)	578 (43.1)	
Clark level			
II	40 (1.1)	63 (4.7)	<.001
III	931 (25.6)	397 (29.6)	
IV	2301 (63.2)	627 (46.7)	
V	358 (9.8)	67 (5.0)	
Not available	10 (0.3)	188 (14.0)	
T category			
T1	403 (11.1)	597 (44.5)	<.001
T2	1511 (41.6)	346 (25.8)	
T3	1149 (31.6)	245 (18.2)	
T4	577 (15.7)	154 (11.5)	
Tumor site			
Trunk	1455 (40.0)	487 (36.3)	<.001
Upper extremity	713 (19.6)	241 (17.9)	
Lower extremity	939 (25.8)	286 (21.3)	
Head and neck	532 (14.6)	326 (24.3)	
Not available	1 (<0.1)	2 (0.2)	
Mitotic rate, per mm^2^			
Range	0-67	0-30	
Mean (SD)	5.74 (6.25)	3.32 (4.17)	
Median (IQR)	4 (2-7)	2 (1-3)	
Mitotic count			
0	259 (7.1)	147 (11.0)	<.001
1	491 (13.5)	186 (13.9)	
>1	2865 (78.7)	536 (39.9)	
Not available	25 (0.7)	473 (35.2)	
Melanoma subtype			
Acral lentiginous	82 (2.2)	41 (3.0)	<.001
Superficial spreading	1764 (48.5)	266 (19.8)	
Lentigo maligna	55 (1.5)	12 (0.9)	
Desmoplastic	357 (9.8)	79 (5.9)	
Nodular	1203 (33.0)	125 (9.3)	
Other	13 (0.4)	751 (56.0)	
Not available	166 (4.6)	68 (5.1)	

Abbreviations: NA, not available; SLNB, sentinel lymph node biopsy; TIL, tumor-infiltrating lymphocyte.

### Patients

A combined cohort of 7331 patients with clinically localized primary cutaneous melanoma diagnosed between January 1, 2000, and December 31, 2014, was assembled for this analysis from existing databases of 2 institutions (the MIA, or Australian cohort, and California Pacific Medical Center, or US cohort). Criteria used to define eligibility and recommend SLNB included melanomas greater than 1.0 mm thick or 1.0 mm or less in thickness with ulceration or a mitotic rate of 1 per mm^2^ or greater.

### Covariates

Information available on 12 prognostic factors was used for subsequent analyses. These factors were patient age, patient sex, tumor site, tumor thickness, ulceration, mitotic rate, presence of microsatellites, presence of regression, tumor-infiltrating lymphocyte (TIL) grade, presence of lymphatic invasion, tumor type, and Clark level.

### Outcome Measures

We defined effectiveness as the appropriateness of patients selected to undergo SLNB, a staging procedure aiming to identify patients with node-positive melanoma. Thus, effectiveness was increased when whichever biopsies were performed detected more patients with node-positive melanoma. We defined cost as medical resources (including but not restricted to monetary expenditures) used in performing SLNBs. Thus, cost was reduced when fewer procedures were performed to identify patients with node-positive melanoma. We defined cost-effectiveness in terms of 2 outcomes from adopting minimum cutoff probabilities used to select patients to undergo SLNBs, assessed by comparing these outcome measures: actual or expected number of positive SLNBs achieved and required number of procedures performed to achieve that many positive SLNBs.

### Statistical Analysis

PCM^17,18^ generated a probabilistic algorithm to predict SLNB positivity by stratifying each cohort (Australian and US) into 3 risk-defined subgroups based on T category and patient age that provided significant discrimination in each cohort. The composition of each risk subgroup is provided in eMethods in Supplement 1. For each risk subgroup in each cohort, a separate algorithm was produced by multiple logistic regression analysis applied to 12 indices generated for each prognostic factor. We then constructed 2 separate composite prognostic algorithms by merging 3 separate logistic regression outputs obtained from 3 patient-risk subgroups. The final output of each algorithm was an individual patient’s estimated probability of experiencing a positive SLNB outcome. The correct discrimination of each composite PCM-generated algorithm was assessed by comparing its AUROC with the AUROC achieved by analyzing the same 12 factors via conventional logistic regression. Prognostic accuracy was assessed by matched-pair analyses of probabilistic prediction errors using Wilcoxon matched-pairs, signed-rank test, and the binomial sign test (eMethods in Supplement 1). All reported *P* values are 2-sided; statistical significance was defined as *P* ≤ .05.

Probabilistic algorithms generated by 3 different methodologies were applied to the 5989-patient Australian cohort: the PCM-generated algorithm, an algorithm derived from conventional multiple logistic regression analysis, and an algorithm combining useful features of both methodologies (eMethods in Supplement 1). Selection of each eligible patient to undergo SLNB was simulated when that patient’s SLNB-positive probability equaled or exceeded a range of minimally acceptable cutoff probabilities. The resulting cost-effectiveness of basing patient selection on different cutoffs was calibrated in terms of 2 outcome measures: actual or expected number of positive SLNBs achieved and the required number of procedures performed to achieve that number. Cost-effectiveness was separately assessed using SLNB-positivity probabilities generated by each of the 3 algorithms produced by each methodology, respectively. The goodness of fit of each algorithm’s probabilities was also assessed by comparing the observed number of positive SLNBs with its expected number, and corresponding *R*^2^ values were calculated. Data were analyzed using SPSS statistical software version 27 (IBM) from October 2000 to April 2021.

## Results

A cohort of 5989 patients at MIA was examined, and 3640 of these patients (2212 males [60.8%]; 2447 aged >50 years [67.2%]) underwent SLNB (Table 1). We also examined 2349 patients at MIA who were eligible for but did not undergo SLNB (eTable 1 in Supplement 1). Separately, 1342 US patients with primary melanoma undergoing SLNB were studied (774 males [57.7%]; 885 aged >50 years [66.0%]) (Table 1).

**Figure 1.**
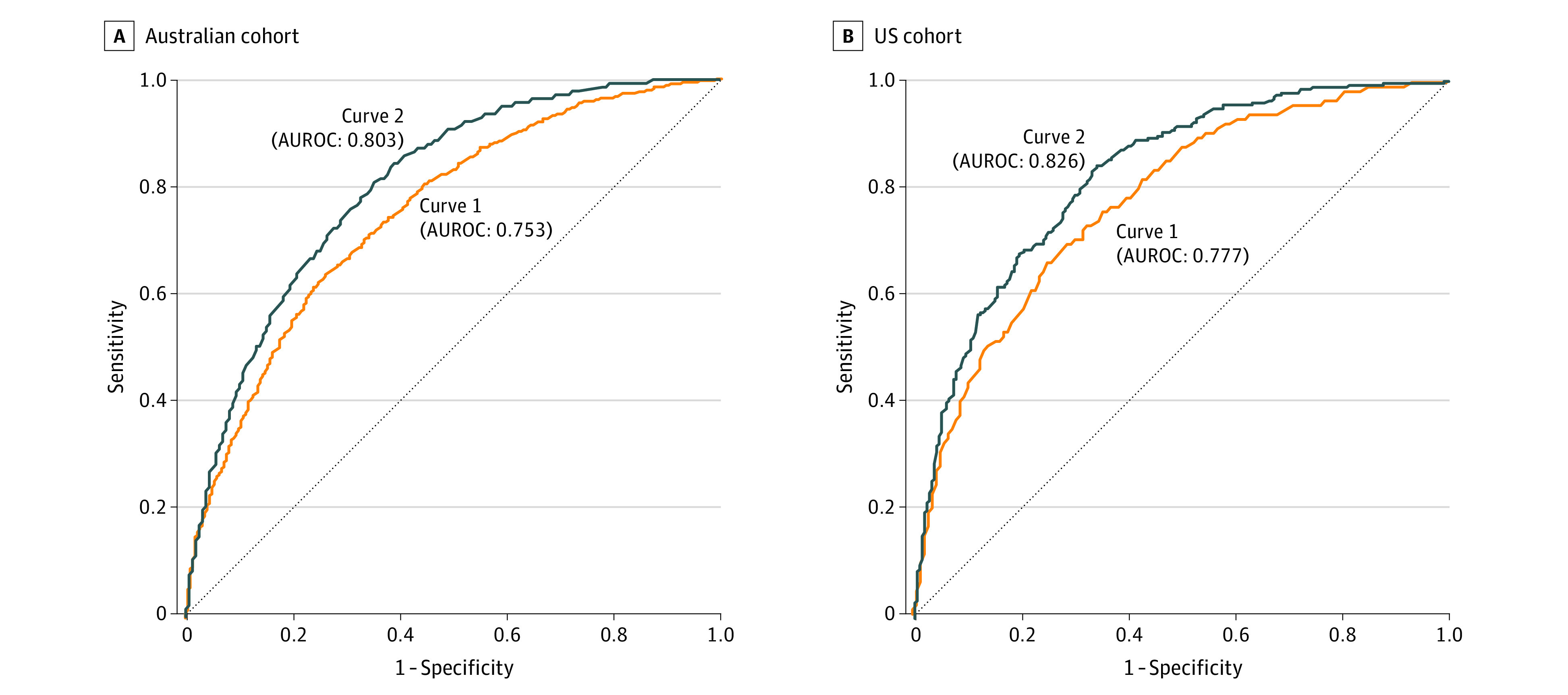
Discriminating Among Sentinel Lymph Node Biopsy Outcomes Using Various Prognostic Methodologies Receiver operating characteristic curve plots of sentinel lymph node biopsy outcome discrimination using conventional logistic regression methodology (curve 1) vs patient-centered methodology (curve 2) are presented in the A, Australian cohort and B, US cohort. AUROC indicates area under the receiver operating characteristic curve.

Initially, we assessed the utility of 12 prognostic factors in estimating individual patient SLNB-positivity probabilities in the Australian cohort using conventional multiple logistic regression. In stepwise analysis of these factors, all except sex were independently associated with SLNB outcome; χ^2^ ranged from 3.88 (*P* = .05) for mitotic rate to 65.14 (*P* < .001) for melanoma subtype (Table 2), with a combined AUROC of 0.753 (Figure 1A).

**Table 2.** Conventional Analysis of Expected Impact of Factors on SLNB Positivity^a^

Covariate	χ^2^^b^	*P* value
Melanoma subtype	65.14	<.001
Thickness	61.73	<.001
Lymphatic invasion	55.98	<.001
Age	51.08	<.001
Regression	50.96	<.001
Clark level	13.09	<.001
Microsatellites	12.01	<.001
Ulceration	11.72	<.001
Tumor site	11.5	<.001
TIL grade	6.78	.009
Mitotic rate	3.88	.05

Abbreviations: SLNB, sentinel lymph node biopsy; TIL, tumor-infiltrating lymphocyte.

^a^ Conventional analysis consisted of stepwise multiple logistic regression with backward elimination. Analysis was among 3640 patients in the Melanoma Institute Australia cohort.

^b^ χ^2^ values indicate deviations separating each regression-estimated numeric coefficient characterizing, respectively, the association of each independent variable with the single dependent variable from its uniformly null-hypothesis–stipulated value of zero. Each tabled *P* value corresponds to its corresponding null-hypothesized zero-value statistical test.

Next, we used PCM to produce comparable probabilities of SLNB positivity. We stratified the Australian cohort into 3 prognostic subgroups based on tumor thickness and patient age, 2 covariates associated with the greatest changes in SLNB outcome (Table 2); 79 of 893 patients in the low-risk group (8.9%) were SLNB positive. This increased to 239 of 1355 patients in the intermediate-risk group (17.6%) and 461 of 1392 patients in the high-risk group (33.1%), with significant differences in SLNB-positivity rates by group as shown by χ^2^ test (*P* < .001) (eTable 2 in Supplement 1).

We then fitted by separate logistic regression analysis an algorithm that best predicted SLNB positivity from the same 12 prognostic factors to each patient subgroup. Distinct subsets of factors were identified as independent covariates in each subgroup (eTables 3-5 in Supplement 1). Prognostic probabilities from each subgroup were merged into a composite index, which achieved an AUROC of 0.803 (Figure 1A). To verify PCM’s increased accuracy, matched pairs of absolute value error differences in predicting SLNB outcomes were assessed, with significant differences favoring smaller PCM errors in the Wilcoxon matched-pair signed-ranks test and the binomial sign test (*P* < .001). PCM produced a more accurate prognosis 69.4% more frequently than when using conventional logistic regression analysis.

Separately, we examined US patients who had undergone SLNB. There were statistically significant differences in the composition of the 2 cohorts (Table 1), with the Australian cohort exhibiting a higher proportion of patients with thicker melanomas and a higher SLNB-positivity rate. In addition, 3 prognostic factors (regression, melanoma subtype, and TIL grade) were coded in different ways, precluding simple merging of data from cohorts and complicating the application of data from 1 cohort to the other. We assessed the prognostic utility of the same 12 prognostic factors with conventional logistic regression analysis, which achieved an AUROC of 0.777 (Figure 1B). As in our analysis of the Australian cohort, we used PCM to generate low-, intermediate-, and high-risk subgroups, with 38 of 597 patients (6.4%), 87 of 458 patients (19.0%), and 112 of 287 patients (39.0%) experiencing a positive SLNB outcome, respectively, with significant differences by group as shown in the χ^2^ test (*P* < .001) eTable 2 in Supplement 1). After merging of probabilities for 3 subgroups, the composite index achieved an AUROC of 0.826 (Figure 1B). The improved accuracy of PCM in predicting SLNB outcomes compared with conventional logistic regression analysis was supported by a matched-pairs analysis of absolute errors (*P* < .001 for signed rank and binomial tests). PCM produced a more accurate prognosis 100.9% more frequently than conventional analysis.

We then assessed the cost-effectiveness of selecting patients to undergo SLNB by virtue of equaling or exceeding a minimum cutoff probability. Separate SLNB-positivity probabilities were generated by 3 separate algorithms. To the 3640 patients in the Australian cohort, we added 2349 patients (eTable 1 in Supplement 1) who were eligible for but did not undergo SLNBs. Probabilities were produced for all 5989 Australian patients from the 2 corresponding algorithms derived from the 3640 Australian patients and 1342 US patients who underwent SLNB. An adequate sample of eligible patients who did not undergo the procedure could not be identified in the US cohort, precluding a similar analysis for that cohort.

**Figure 2.**
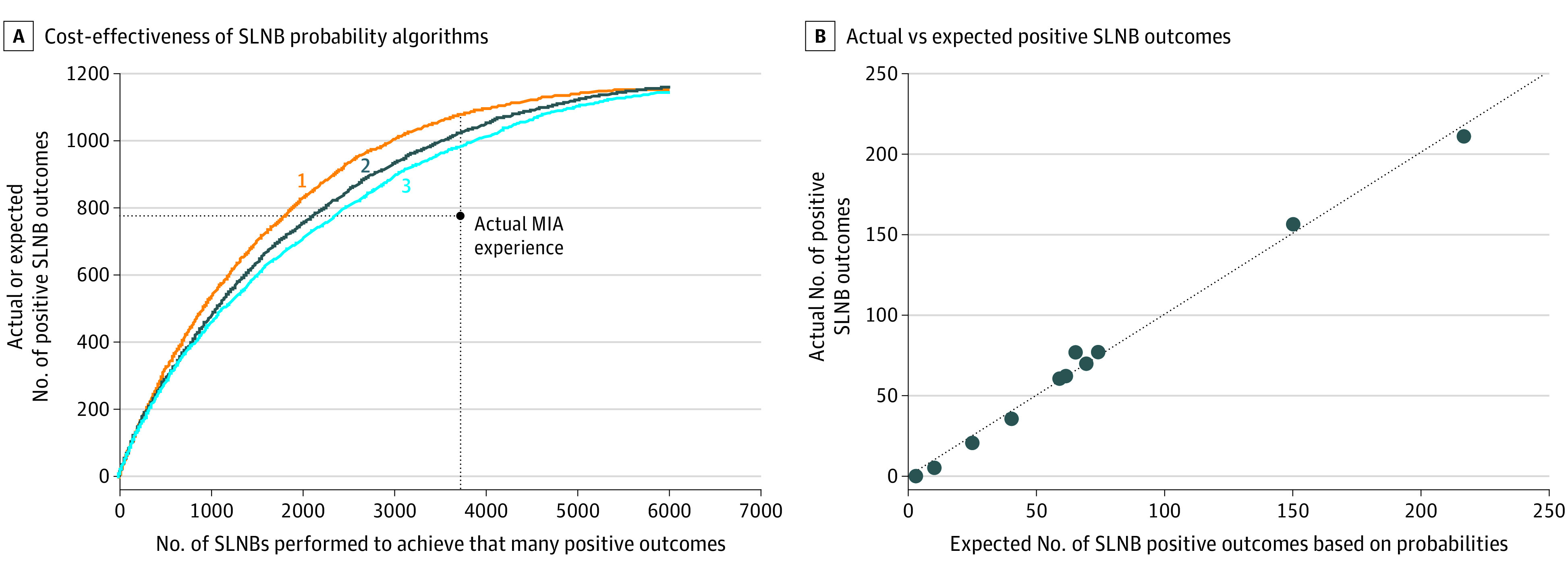
Cost-effectiveness of Applying Various Algorithms and Concordance Between Expected and Actual Outcomes A, A comparison is presented of the cost-effectiveness of various sentinel lymph node biopsy (SLNB)–positive probability algorithms used to guide simulated selection of patients to undergo SLNB, all applied to the Melanoma Institute Australia (MIA) cohort. Curve 1 applies the patient-centered methodology–generated algorithm from the Australian cohort, curve 2 applies the algorithm developed using conventional methodology from the Australian cohort, and curve 3 applies the SLNB outcome algorithm from the US cohort to the same patients in the Australian cohort. B, Concordance is presented between patient-centered methodology–generated expected counts of positive SLNB outcomes (based on patient-centered methodology–generated probabilities) and the actual recorded counts of positive SLNB outcomes.

Using the PCM-generated algorithm, adopting an 8.7% minimum cutoff probability of SLNB positivity resulted in the same number of simulated SLNBs as actually performed (3640 SLNBs) but with 1066 expected positive outcomes, for a positivity rate of 29.3%. This constituted an improvement of 287 SLNBs compared with 779 actual positive SLNBs (36.8% improvement). In contrast, adopting a 23.7% minimum cutoff resulted in performing 1825 simulated SLNBs, with the same expected number of positive outcomes (779 SLNBs), for a positivity rate of 42.7%, and requiring 1815 fewer simulated procedures (49.9%). Each minimum cutoff probability that produced curve 1 of Figure 2A between the previously described 2 reference probabilities illustrated cost-effective dominance (ie, larger numbers of expected positive SLNB outcomes and smaller numbers of procedures required to achieve these positive outcomes compared with the actual experience).

In addition, we assessed results when applying 4 PCM-generated minimum-cutoff SLNB–positivity reference probabilities that may be applied in practice (range, 5%-20%). Melanoma centers typically recommend SLNB to an individual patient when the probability of a positive outcome ranges between 5% and 10%. Adopting 10%, 15%, or 20% PCM-generated minimum cutoffs improved both outcome measures simultaneously (given that they were within the cost-effective dominance range of 8.7% and 23.7%) (Table 3). For example, the PCM cutoff of 10% produced 1046 expected positive SLNBs of 3417 simulated SLNBs performed (36.0%), constituting an improvement of 267 more than the 779 positive SLNBs actually achieved (34.3% improvement) and a reduction of 223 unneeded SLNBs from the 3640 procedures actually performed (6.1% reduction). In contrast, adopting the 5% minimum cutoff probability increased the number of expected positive SLNB outcomes, resulting in a SLNB-positivity rate of 1123 of 4636 SLNBs (24.2%), for an increase of 344 positive SLNBs (44.2%); however, it required 996 additional expected procedures to be performed(27.4%) (Table 3). All possible PCM-generated minimum cutoff probabilities and associated pairs of SLNB outcomes are provided in eTable 6 in Supplement 2, ranked by ascending positivity probabilities.

**Table 3.** Simulated Impact on Cost-effectiveness of Using Different Methodologies to Select Patients to Undergo SLNB vs Actual Experience

Minimum positivity cutoff probability	SLNBs performed, No.	Positive SLNBs, No.	Efficiency, % positivity	No. (%)	
				Increase in positivity	Reduction in SLNBs
Actual experience in MIA cohort	3640	779	21.40	NA	NA
PCM algorithm applied to Australian cohort^a^					
0.050	4636	1123	24.20	344 (44.2)	−996 (−27.4)
0.087	3640	1066	29.30	287 (36.8)	0
0.100	3417	1046	30.60	267 (34.3)	223 (6.1)
0.150	2623	952	36.30	173 (22.2)	1017 (27.9)
0.200	2092	844	40.30	65 (8.3)	1548 (42.5)
0.237	1825	779	42.70	0	1815 (49.9)
Conventional algorithm applied to Australian cohort^b^					
0.050	5250	1130	21.50	351 (45.1)	−1610 (−44.2)
0.100	3968	1044	26.30	265 (34.0)	−328 (−9.0)
0.113	3640	1011	27.80	232 (29.8)	0
0.150	2830	904	31.90	125 (16.1)	810 (22.3)
0.198	2134	779	36.50	0	1506 (41.4)
0.200	2117	775	36.60	−4 (−0.5)	1523 (41.8)
Mixed algorithm from US applied to Australian cohort^c^					
0.050	5128	1104	21.50	325 (41.7)	−1488 (−40.9)
0.100	3721	979	26.30	200 (25.7)	−81 (−2.2)
0.105	3640	972	26.70	193 (24.8)	0
0.150	2722	841	30.90	61 (7.8)	918 (25.2)
0.174	2380	779	32.70	0	1260 (34.6)
0.200	2030	712	35.10	−67 (−8.6)	1610 (44.2)

Abbreviations: MIA, Melanoma Institute Australia; NA, not applicable; PCM, patient-centered methodology; SLNB, sentinel lymph node biopsy.

^a^ Probabilities are listed in eTable 6 in Supplement 2. Minimum cutoff probabilities between 0.087 and 0.237 were cost-effective dominant vs the actual experience (ie, there were nonnegative numbers for increased positivity and reduced SLNBs).

^b^ The conventional algorithm was a logistic regression–generated algorithm. Probabilities are listed in eTable 7 in Supplement 2. Minimum cutoff probabilities between 0.113 and 0.198 were cost-effective dominant vs the actual experience (ie, there were nonnegative numbers for increased positivity and reduced SLNBs).

^c^ The mixed algorithm was a PCM and conventional logistic regression–generated algorithm. Probabilities are listed in eTable 8 in Supplement 2. Minimum cutoff probabilities between 0.105 and 0.174 were cost-effective dominant vs the actual experience (ie, there were nonnegative numbers for increased positivity and reduced SLNBs).

We next assessed the cost-effectiveness of judicious patient selection when cutoff probabilities were generated from conventional multiple logistic regression analysis of the same 12 prognostic factors in the MIA cohort. Use of these probabilities in simulation also resulted in improved cost-effectiveness compared with actual experience, although with smaller differences than found when using probabilities generated by PCM (Figure 2A). The range of cost-effective dominant minimum cutoff probabilities decreased to between 11.3% and 19.8%. Thus, 5%, 10%, and 20% reference cutoffs were outside the dominance range (Figure 2A and Table 3) and therefore did not generate unambiguously superior selections. For example, the conventional cutoff of 10% resulted in an expected positivity rate of 1044 of 3968 SLNBs performed (26.3%), constituting an improvement of 265 positive SLNBs vs the actual experience (34.0% improvement) but an increase of 328 unneeded SLNBs (9.0%). All possible minimum cutoff probabilities and associated pairs of SLNB outcomes generated by this analysis are provided in eTable 7 in Supplement 2.

Despite significant differences in composition of the 2 cohorts, we sought to investigate whether a probability-estimating algorithm derived from 1 cohort could improve the cost-effectiveness of selecting patients in the other cohort. A prognostic algorithm was produced from the US cohort by combining PCM and conventional logistic regression analysis. Application of this algorithm to the 5989-patient MIA cohort also improved the cost-effectiveness of selecting patients to undergo SLNB compared with actual experience (Figure 2A), albeit with a smaller improvement than with other algorithms. Specific outcomes achieved using the same reference minimum cutoff probabilities are shown in Table 3. For example, adopting a 10.5% minimum cutoff probability resulted in performing 3640 simulated SLNBs, the same number as actually performed, with 193 additional expected positive outcomes more than the 779 actually achieved (a 24.8% increase), for a positivity rate of 26.7%. All possible minimum cutoff probabilities and associated pairs of SLNB outcomes appear in eTable 8 in Supplement 2.

Finally, comparison of the observed number of positive SLNBs with their corresponding expected values revealed a close correspondence. Uncorrected *R*^2^ values were 0.993 for PCM (Figure 2B), 0.978 for the conventional methodology (eFigure in Supplement 1), and 0.958 for the mixed PCM and conventional methodology (eFigure 1 in Supplement 1).

## Discussion

Melanoma is expected to represent the second most common malignant tumor in the US by 2040.^19^ An estimated mean cost of more than $25 000^20^ per SLNB in the US translates to substantial current and anticipated future costs. Given that approximately 80% of patients who currently undergo SLNB do not have lymph node metastases, selecting appropriate patients for SLNB in a cost-effective manner may become increasingly important and worthwhile.

This hybrid prognostic study/decision analytical model found improved discrimination in predicting SLNB positivity. These results extend prior such attempts. Investigators at the Memorial Sloan Kettering Cancer Center developed a nomogram using 5 prognostic factors that achieved an AUROC of 0.694.^15^ More recently, Lo and colleagues at Melanoma Institute Australia^16^ developed a nomogram using 6 prognostic factors and reported an AUROC of 0.739. The incorporation of gene expression–profiling assays may be associated with further improvement in prognostic discrimination, but to date no detailed comparative studies have been reported.^21,22^ The greater prognostic discrimination achieved in our study (AUROCs of 0.826 and 0.803 in US and Australian cohorts, respectively) was realized by using PCM to estimate a separate, individually and institutionally tailored probability of SLNB positivity based on 12 established and routinely available clinical and histopathological prognostic factors. Several features of PCM enabled its increased capability in our models,^17,18^ including stratifying the population into more homogeneous risk subgroups, developing indices for each prognostic factor reflecting the shape of its impact on the outcome measure of interest, and special handling of missing observations.

The improved prognostic capability of PCM was exploited to enable improvements in the cost-effectiveness of selecting patients to undergo SLNB. Patients were selected by comparing their estimated SLNB-positive probability with a minimum cutoff probability. The PCM-based selection procedure produced an expanded range of cost-effectiveness dominance compared with that obtained using conventional logistic regression analysis. Both methodologies improved cost-effectiveness compared with the actual experience in the Australian cohort. Intriguingly, using a 5% cutoff probability (which is used by many melanoma centers) resulted in outcomes that were outside the PCM cost-effective dominance range, whereas using a 10% value produced outcomes within the range. This suggests that appropriate minimum cutoff probabilities recommended by various guidelines may usefully be revisited.^23^

Our results suggest substantial room for improvement in the cost-effectiveness of the process by which patients with melanoma are currently selected to undergo SLNB. To achieve this improvement, each health system may need to choose its preferred minimum cutoff probability, with the goal of reducing the total number of patients undergoing SLNB (by up to 49.9% in the Australian cohort) or increasing the number of patients with node-positive melanoma detected (by up to 36.8% in this cohort) or some balance between these goals. Minimum cutoff probabilities and resulting pairs of SLNB outcomes indicated the consequences of applying the chosen cutoff probability using each algorithm, while outcomes found using cutoff probabilities and cost-effectiveness curves indicated whether that probability was within each algorithm’s cost-effective dominance range.

An important possibility is that with a sufficiently large sample of patients required to generate stable probabilistic estimates, each health system may develop an algorithm tailored to the composition of its own patient population and reflecting the distinct ways various prognostic factors are recorded. Any such locally tailored (or externally obtained) algorithm may be incorporated into an interactive electronic (eg, web-based) tool whose inputs are specific prognostic factors and whose outputs are calculated SLNB-positive probabilities for local individual patients. We recommend that the National Comprehensive Cancer Network and other national melanoma guideline committees consider these issues in discussing minimum cutoff probabilities. At the individual patient level, this may have important implications for the decision-making process. For example, the conversation with a male patient aged 34 years with a 0.7-mm acral melanoma without TILs (harboring >10% risk) would be markedly different than that with a female patient aged 87 years with a 1.8-mm desmoplastic melanoma on the upper arm (harboring <5% risk).

### Limitations

This study has several limitations, including compositional differences between cohorts and the difficulty in applying an algorithm from 1 institution to another when relevant prognostic factors were not similarly coded. This analysis constitutes a retrospective simulation demonstrating the potentially improved cost-effectiveness achievable by selecting various cutoff probabilities. However, a prospective clinical trial would be required to confirm improvements realized by adopting a chosen minimum cutoff probability to select patients for SLNB using PCM or any other appropriate prognostic methodology.

## Conclusions

Beyond SLNB for melanoma, results of this hybrid prognostic study/decision analytical model study may have important implications for how guidelines are developed for selecting patients to undergo other useful medical procedures. These results suggest the consequences that choosing different minimum cutoff probabilities may have for the cost-effectiveness of the selection process and the unambiguous advantages that may be gained by selecting patients within the cost-effective dominance range when one is identified. Importantly, this approach reflects a conceptual advance beyond a strictly categorical approach (ie, whether a patient is eligible to undergo a given procedure) to a comparative approach (ie, which patients are more eligible to undergo that procedure) in contexts in which differing degrees of eligibility are sensible and ascertainable.

## Notes

Supplement 1.
eMethods.

eReferences.
**eFigure.** Concordance Between Expected Positive Outcomes vs Actual Counts **eTable 1.** Characteristics of Australian Patients Eligible for But Not Receiving Sentinel Lymph Node Biopsy **eTable 2.** Defining Characteristics of Risk Subgroups and Prevalence of Sentinel Lymph Node Biopsy Positivity **eTable 3.** Stepwise Multiple Logistic Regression of Impact of Factors on Sentinel Lymph Node Biopsy Positivity in 893 Patients in Australian Low-Risk Subset **eTable 4.** Stepwise Multiple Logistic Regression of Impact of Factors on Sentinel Lymph Node Biopsy Positivity in 1355 Patients in Australian Intermediate-Risk Subset **eTable 5.** Stepwise Multiple Logistic Regression of Impact of Factors on Sentinel Lymph Node Biopsy Positivity in 1392 Patients in Australian High-Risk Subset

Supplement 2. **eTable 6.** Patient-Centered Methodology–Generated Minimum Cutoff Probabilities and Associated Pairs of Patient Selection Outcomes in Australian Cohort **eTable 7.** Minimum Cutoff Probabilities Generated by Conventional Analysis and Associated Pairs of Patient Selection Outcomes in Australian Cohort **eTable 8.** Minimum Cutoff Probabilities Generated by Mixed Analysis and Associated Pairs of Patient Selection Outcomes in Australian Cohort

Supplement 3.
Data Sharing Statement
