# Supplementary material for: Improving Selection for Sentinel Lymph Node Biopsy Among Patients With Melanoma
Source: JAMA Netw Open. 2023 Apr 19;6(4):e236356. doi: 10.1001/jamanetworkopen.2023.6356 (PMC10116363; doi:10.1001/jamanetworkopen.2023.6356)
Supplement: Supplement 1. — eMethods. eReferences. eFigure. Concordance Between Expected Positive Outcomes vs Actual Counts eTable 1. Characteristics of Australian Patients Eligible for But Not Receiving Sentinel Lymph Node Biopsy eTable 2. Defining Characteristics of Risk Subgroups and Prevalence of Sentinel Lymph Node Biopsy Positivity eTable 3. Stepwise Multiple Logistic Regression of Impact of Factors on Sentinel Lymph Node Biopsy Positivity in 893 Patients in Australian Low-Risk Subset eTable 4. Stepwise Multiple Logistic Regression of Impact of Factors on Sentinel Lymph Node Biopsy Positivity in 1355 Patients in Australian Intermediate-Risk Subset eTable 5. Stepwise Multiple Logistic Regression of Impact of Factors on Sentinel Lymph Node Biopsy Positivity in 1392 Patients in Australian High-Risk Subset [file jamanetwopen-e236356-s001.pdf]

Supplemental Online Content

Miller JR III, Lo SN, Nosrati M, et al. Improving selection for sentinel lymph node biopsy among patients with melanoma. *JAMA Netw Open*. 2023;6(4):e236356.
doi:10.1001/jamanetworkopen.2023.6356

eMethods
eReferences
eFigure. Concordance Between Expected Positive Outcomes vs Actual Counts
eTable 1. Characteristics of Australian Patients Eligible for But Not Receiving Sentinel Lymph Node Biopsy
eTable 2. Defining Characteristics of Risk Subgroups and Prevalence of Sentinel Lymph Node Biopsy Positivity
eTable 3. Stepwise Multiple Logistic Regression of Impact of Factors on Sentinel Lymph Node Biopsy Positivity in 893 Patients in Australian Low-Risk Subset
eTable 4. Stepwise Multiple Logistic Regression of Impact of Factors on Sentinel Lymph Node Biopsy Positivity in 1355 Patients in Australian Intermediate-Risk Subset
eTable 5. Stepwise Multiple Logistic Regression of Impact of Factors on Sentinel Lymph Node Biopsy Positivity in 1392 Patients in Australian High-Risk Subset

This supplemental material has been provided by the authors to give readers additional information about their work.

© 2023 Miller JR III et al. *JAMA Network Open.*

eMethods

To develop PCM-generated algorithms,[1-3] three risk subgroups were identified in each cohort (summarized in Table S2). In the Australian cohort, this comprised a low-risk subgroup with Breslow thickness (BT) $<$1.3 mm; an intermediate-risk subgroup either with BT 1.3-1.8 mm or with BT 1.8-4.0 mm and age $\geq$62; and a high-risk subgroup either with BT 1.8-4.0 mm and age $<$62 or with BT $\geq$4.0 mm. In the U.S. cohort, this comprised a low-risk subgroup with BT $\leq$1.0 mm; an intermediate-risk subgroup either with BT 1.0-2.0 mm or with BT 2.0-4.0 mm and age $\geq$ 62; and a high-risk subgroup either with BT 2.0-4.0 mm and age $<$62 or with BT $>$4.0 mm.

For the matched-pair analysis, each of the 3,640 patients was assigned a pair of probabilistic estimates of SLNB-positivity, generated by PCM and by conventional methodology. Each probability was subtracted from the patient's known outcome (coded as 1 for positive or 0 for negative), and the matched pairs of absolute-value error differences were tested via the Wilcoxon matched-pairs, signed ranks test and the binomial sign test.

The third prognostic algorithm was generated using multiple logistic regression analysis of 11 prognostic factors in the U.S. cohort, and applied to the Australian cohort. It utilized PCM-generated indexes for patient age, tumor site, and mitotic rate, supplemented by conventional indexes developed for tumor-infiltrating lymphocyte (TIL) grade[4] and melanoma subtype. No partitioning into risk subgroups was performed due to substantial differences in median tumor thickness between the two cohorts. Tumor regression was eliminated from this algorithm, due to the substantially different coding of this factor between the two cohorts.

© 2023 Miller JR III et al. *JAMA Network Open.*

eReferences

1. Miller JR III, Kashani-Sabet M, Sagebiel RW. Patient-centered prognosis. A methodology to improve individually tailored prognostic accuracy illustrated in two cancers. iUniverse. 2013.

2. Miller JR III, Kashani-Sabet M, Sagebiel RW. Patient-centered diagnosis. Methodologies to predict individually tailored outcomes of current diagnostic tests and to create new tests. iUniverse, 2015.

3. Miller JR III, Kashani-Sabet M, Sagebiel RW. Patient-centered cure assessment. A methodology to assess whether medical interventions succeed in curing individual patients. iUniverse, 2017.

4. Azimi F, Scolyer RA, Rumcheva P, et al. Tumor-infiltrating lymphocyte grade is an independent predictor of sentinel lymph node status and survival in patients with cutaneous melanoma. *J Clin Oncol.* 2012;30:2678-83.

© 2023 Miller JR III et al. *JAMA Network Open.*

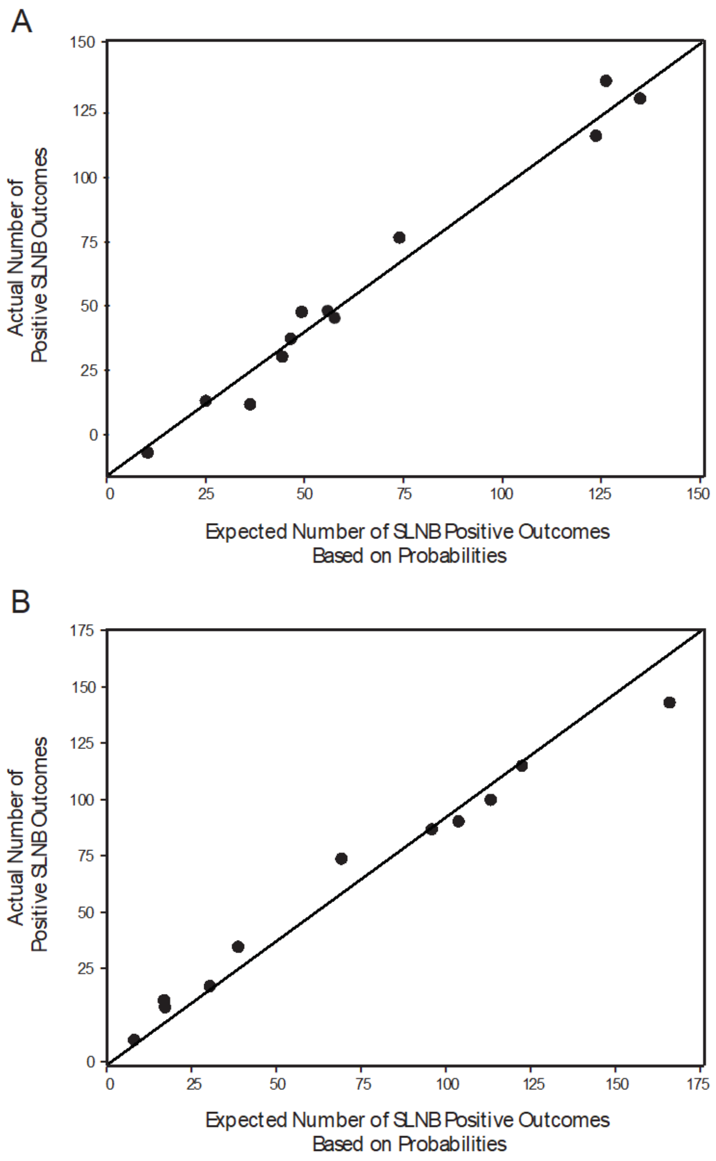

eFigure. Concordance Between Expected Positive Outcomes vs Actual Counts

Panel A. Concordance between expected counts of SLNB-positive outcomes generated by conventional estimating methodology (logistic regression) applied to the Australian cohort and recorded actual counts in that cohort. Panel B refers to the concordance between actual counts in the Australian cohort and expected counts estimated from patient data in the U.S. cohort and then applied to patients in the Australian cohort.

© 2023 Miller JR III et al. *JAMA Network Open.*

eTable 1. Characteristics of Australian Patients Eligible for But Not Receiving Sentinel Lymph Node Biopsy

	(N=2,349)
Male gender	1,379 (58.7%)
Age > 50 years	1,773 (75.5%)
Ulceration present	544 (23.2%)
TIL grade	
0	1,086 (46.2%)
1-3	1,263 (53.8%)
Regression	
Late	300 (12.8%)
Absent	1,824 (77.6%)
Intermediate	225 (9.6%)
Lymphatic invasion	
No	2,155 (91.8%)
Yes	116 (4.9%)
N/A	78 (3.3%)
Microsatellites	
No	1,972 (84.0%)
Yes	104 (4.4%)
N/A	273 (11.6%)
Clark level	
II	211 (9.0%)
III	847 (36.0%)
IV	1,014 (43.2%)
V	266 (11.3%)
N/A	11 (0.5%)

© 2023 Miller JR III et al. *JAMA Network Open.*

T category	
T1	953 (40.6%)
T2	570 (24.3%)
T3	409 (17.4%)
T4	417 (17.7%)
Tumor site	
Trunk	791 (33.7%)
Upper extremity	464 (19.8%)
Lower extremity	555 (23.6%)
Head and neck	534 (22.7%)
N/A	5 (0.2%)
Mitotic rate (per mm^2)	
Range	0-98
Mean	4.79
Median	2
0	132 (5.6%)
1	707 (30.1%)
>1	1,499 (63.8%)
N/A	11 (0.5%)
Melanoma subtype	
Acral lentiginous	50 (2.1%)
Superficial spreading	1,346 (57.3%)
Lentigo maligna	101 (4.3%)
Desmoplastic	205 (8.7%)
Nodular	511 (21.8%)
Other	9 (0.4%)
N/A	127 (5.4%)

© 2023 Miller JR III et al. *JAMA Network Open.*

eTable 2. Defining Characteristics of Risk Subgroups and Prevalence of Sentinel Lymph Node Biopsy Positivity

**Cohort**	**Risk Subgroup**		
	Low-Risk	Intermediate-Risk	High-Risk
MIA	BT <1.3 (8.85%)	[BT 1.3-1.8] or [BT 1.8-4.0 & age≥62] (17.6%)	[BT 1.8-4.0 & age<62] or [BT >4.0] (33.1%)
U.S.	BT <1.0 (6.4%)	[BT 1.0-2.0] or [BT 2.0-4.0 & age≥62] (19.0%)	[BT 2.0-4.0 & age<62] or [BT >4.0] (39.0%)

BT- Breslow thickness (in mm).

© 2023 Miller JR III et al. *JAMA Network Open.*

eTable 3. Stepwise Multiple Logistic Regression of Expected Impact of Factors on Sentinel Lymph Node Biopsy Positivity in 893 Patients in Australian Low-Risk Subset

Predictor	Chi-square	P value
Age	26.92	< 0.001
Mitotic rate	10.07	0.002
Regression	9.66	0.002
Melanoma subtype	6.66	0.01
TIL grade	5.03	0.03
Tumor site	4.28	0.04
Thickness	3.76	0.05
Lymphatic invasion	3.73	0.05

© 2023 Miller JR III et al. *JAMA Network Open.*

eTable 4. Stepwise Multiple Logistic Regression of Expected Impact of Factors on Sentinel Lymph Node Biopsy Positivity in 1355 Patients in Australian Intermediate-Risk Subset

Predictor	Chi-square	P value
Melanoma subtype	32.30	< 0.001
Regression	19.51	< 0.001
Tumor site	18.38	< 0.001
Mitotic rate	15.98	< 0.001
Microsatellites	12.94	0.003
Age	12.55	0.004
Lymphatic invasion	11.91	0.006
Clark level	10.77	0.001
Thickness	4.42	0.04
TIL grade	4.19	0.04

© 2023 Miller JR III et al. *JAMA Network Open.*

eTable 5. Stepwise Multiple Logistic Regression of Expected Impact of Factors on Sentinel Lymph Node Biopsy Positivity in 1392 Patients in Australian High-Risk Subset

Predictor	Chi-square	P value
Melanoma subtype	62.18	< 0.001
Regression	33.20	< 0.001
Lymphatic invasion	24.56	< 0.001
Age	13.06	< 0.001
Tumor site	11.26	< 0.001
Thickness	10.72	0.001
TIL grade	8.32	0.004
Mitotic rate	7.82	0.005
Ulceration	4.12	0.04

© 2023 Miller JR III et al. *JAMA Network Open.*
