# Supplementary material for: Improving Selection for Sentinel Lymph Node Biopsy Among Patients With Melanoma
Source: JAMA Netw Open. 2023 Apr 19;6(4):e236356. doi: 10.1001/jamanetworkopen.2023.6356 (PMC10116363; doi:10.1001/jamanetworkopen.2023.6356)
Supplement: Supplement 3. — Data Sharing Statement [file jamanetwopen-e236356-s003.pdf]

Data Sharing Statement

Miller. Improving Selection for Sentinel Lymph Node Biopsy Among Patients With Melanoma. *JAMA Network Open*. Published online April 19, 2023. doi:10.1001/jamanetworkopen.2023.6356

Data
Data available: No

Additional Information
Explanation for why data not available: The data will be made available upon request.

© 2023 Miller JR III et al. *JAMA Network Open*.
